# A parameter optimization algorithm for intensity‐modulated radiotherapy prostate treatment planning*

**DOI:** 10.1120/jacmp.v3i3.2567

**Published:** 2002-06-01

**Authors:** J. Barbiere, M. F. Chan, J. Mechalakos, D. Cann, K. Schupak, C. Burman

**Affiliations:** ^1^ Department of Medical Physics Memorial Sloan‐Kettering Cancer Center 23 Pocono Rd. Denville New Jersey 07834

**Keywords:** IMRT, optimization, prostate cancer, radiotherapy

## Abstract

An iterative algorithm has been developed to analytically determine patient specific input parameters for intensity‐modulated radiotherapy prostate treatment planning. The algorithm starts with a generic set of inverse planning parameters that include dose and volume constraints for the target and surrounding critical structures. The overlap region between the target volume and the rectum is used to determine the optimized target volume coverage goal. Sequential iterations are performed to vary the numerous parameters individually or in sets while other parameters remain fixed. A coarse grid search is first used to avoid convergence on a local maximum. Linear interpolation is then used to define a region for a fine grid search. Selected parameters are also tested for possible improvements in target coverage. In several representative test cases investigated the coverage of the planning target volume improved with the use of the algorithm while still meeting the clinical acceptability criteria for critical structures. The algorithm avoids time‐consuming random trial and error variations that are often associated with difficult cases and also eliminates lengthy user learning curves. The methodology described in this paper can be applied to any treatment planning system that requires the user to select the input optimization parameters.

PACS number(s): 87.53.–j, 87.90.+y

## INTRODUCTION

A large fraction of the intensity‐modulated radiotherapy (IMRT) planning process involves the optimization stage. Certain algorithms require a set of initial input parameters characterizing the target and surrounding critical structures. Inverse planning algorithms determine the optimized dose distribution by incorporating those input parameters in an objective function and then minimizing the objective function. The end result of optimization is dependent on the user selected input parameters.^1^ ^,^ ^2^

The degree of optimization achieved when the planner selects the input parameters, referred to as “manual planning,” is dependent on the individual planner's experience.^3^ The number of variations in optimization parameters that can be tested is limited by time.^4^ Previous work in automated inverse treatment planning has shown that a single set of inverse planning parameters can generate acceptable plans.^5^

The algorithm presented in this work, referred to as “automated planning,” analytically determines consistent individual input parameters. An orderly series of changes are made on predetermined initial values that can improve target coverage while maintaining the critical structure constraints. Also, instead of relying on generic acceptable target coverage, the algorithm computes a specific goal for individual cases.

## MATERIALS AND METHODS

This work was performed at a regional center of the Memorial Sloan‐Kettering Cancer Center (MSKCC) where prostate patients were planned with a five‐field IMRT technique to 81 Gy.^6^ ^,^ ^7^ The radiation oncologist entered the planning target volume (PTV) and critical structures. The PTV included the prostate and seminal vesicles with a 0.6 cm posterior margin and 1.0 cm margin elsewhere. The IMRT plans were carried out on a planning system developed at the MSKCC. The IMRT option in the planning system uses an inverse planning algorithm to minimize an objective function which is represented as the quadratic sum of squares of the difference between the desired and the actual doses.8 A dose that deviates from the prescribed dose in the PTV or exceeds the constraints in critical structures is subjected to a relative penalty in the objective function. The resultant dose distribution from these intensity‐modulated beams is calculated using a pencil beam algorithm.^9^

IMRT prostate plans require a number of input parameters for the PTV and surrounding critical structures. A prescription dose, minimum desired dose $\left(D_{\text{min}}\right)$, penalty for dose lower than $D_{\min}$, maximum desired dose $\left(d_{\max}\right)$, and penalty for dose higher than $d_{\max}$ are specified for the PTV Input parameters for a critical structure include a dose limit, penalty for dose higher than the dose limit, and volumetric constraints specified as a dose limit, penalty, and volume. Acceptable prostate plans compared in this study had a PTV maximum dose of 110% with at least 90% of the volume treated to 95% of the prescribed dose (i.e., V95>90%). Rectum and bladder were constrained by both maximum dose and volume receiving 50% of the prescribed dose.

The optimization parameter algorithm sets a goal of target coverage that meets and often exceeds the generic acceptable value. Since the PTV includes a target margin that overlaps critical structures, it is impossible and undesirable to expect 100% target coverage with 100% of the prescribed dose. The rectum constraints limit the dose to a portion of the PTV. Increased rectal overlap results in decreased PTV coverage.^210^ The percentage target volume (TF) that does not overlap with the rectum is calculated from the PTV volume (TV) and overlap volume (OV),

TF=100(TV−OV)/TV.

**Figure 1:**
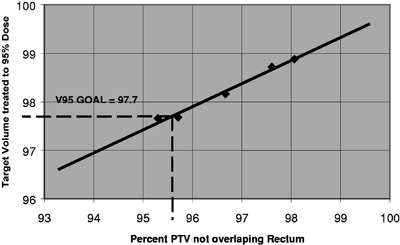
Linear variation of the target volume treated to 95% of the dose (V95) as a function of the percent PTV which does not overlap with the rectum volume (TF). For a patient with TF TF=95.6 the corresponding V95 goal is 97.7.

The algorithm in this study generates the goal V95 as a linear function of TF based on five optimized cases representing the expected TF range. The goal value of V95 is useful in plan evaluation to determine if an effort should be made to improve an otherwise acceptable plan generated by the initial input parameters. For the example shown in Fig. 1, a plan with TF=95.6 has a V95 goal of 97.7. If the initial input parameters result in plan with V95 of 93, which is above the generic acceptable value of 90, manual planning may be terminated. However, automated planning indicates that improved target coverage should be expected and proceeds to generate new input parameters.

The algorithm separates the input parameters into four categories. The first type of input parameter, including all the overlap region dose constraints, does not change from the initial value shown in Table I. These constant parameters serve as a basis for normalizing the remaining variables in the objective functions.

**Table I:** Initial input parameters for MSKCC prostate treatment planning to 8100cGy. All the “Target and rectum” overlap parameters and “Target not rectum” dose, $d_{\min},d_{\max}$ shown in gray are kept constant.

	Dose	$d_{\text{min}}$	Penalty	$d_{\max}$	Penalty
Target not rectum	100	98	50	102	100
Target and rectum	96	93	10	96	20
	Dose limit	Penalty	Dose limit	Penalty	Volume
Rectum	96	20	40	20	70
Bladder	100	5	40	20	70

The second type of input parameter consists of parameters that are varied as a set. The volumetric constraints for the critical structures are varied as a set [dose, penalty, volume]. The critical structure dose constraints are varied as a set [dose limit, penalty]. By varying parameters as a set we are able to keep all variables relatively close to their initial values, which experience has shown to be generally acceptable and also reduce the number of free variables in the search process. The third type of input parameter is that which can be varied independently, such as the target dose [$D_{\text{min}},d_{\max}$] penalties. Finally, the fourth type of input parameter is that which can be disregarded. In cases where there is no significant overlap between the target and the bladder, the bladder parameters can be left out of the optimization process. The decrease in number of variables results in a fewer iterations to complete the optimization process.

Unlike optimization schemes that randomly vary parameters seeking improvements in the outcome as exemplified by the classic simulated annealing,11 this algorithm searches for new values for input parameters in three ways: grid search, linear interpolation, and discrete values. Since experience has shown that the generic initial input parameters shown in Table I produce results in the neighborhood of the final choice, some parameters are varied using a simple grid search. Grid searches are first performed with relatively large changes (coarse grid) to avoid being trapped in a local maximum and are then followed by small changes (fine grid). For the example shown in Table II, if the rectum volume set [dose limit, penalty, volume] of [40,20,70] results in a value 10% above the acceptable limit for the volume of rectum treated to 50% of the prescribed dose (RV50), the next three iterations are performed with changes of 5%, 10%, and 15%, i.e., [38,21,73.5], [36,22,77], and [34,23,80.5]. In order to decrease RV50, the dose limit is decreased while the penalty and volume parameters are increased. If the RV50 result is close to the acceptable limit, then the change required to produce new trial values would be reduced proportionally.

**Table II:** Rectum volume set input parameter grid search to decrease RV50 by 10%.

	Dose limit	Penalty	Volume
Initial value	40	20	70
5% change	38	21	73.5
10% change	36	22	77.0
15% change	34	23	80.5

**Figure 2:**
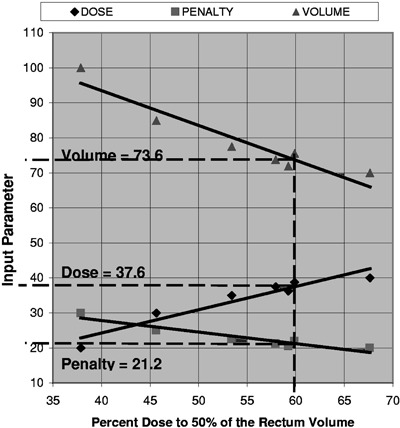
Linear variation of the rectum [dose, penalty, volume] set. Coarse grid, linear interpolation, and fine grid searches determine the optimum input parameters to treat 50% of the rectum volume with 60% for the target dose.

For the cases presented in this work, it has been observed that many outcomes such as the rectum RV50 are linearly dependent on the input parameters [dose, penalty, volume] in the vicinity of acceptable plans. Linear interpolation in two dimensions^12^ using several data points obtained from the coarse grid search is a very good estimate of the final input parameter. The actual value can be found by performing a fine grid search in the vicinity of the interpolated value. Figure 2 shows an example in which the rectum volume input parameters are determined analytically for a desired RV50=60%.

Experience has also shown that some parameters are insensitive to small changes. Instead of using repeated interval iterations, the target dose minimum penalty was chosen to only have the discreet values of 50 or 20. The target dose maximum penalty was chosen to only have the discrete values of 50, 100, or 150.

In order to minimize the effect that changes in one input parameter produce in the overall dose distribution, the optimization is performed in a predefined order. The algorithm sequence used in this work consists of finding parameters for the rectum, bladder, target, and then revising the rectum parameters. Other sequences were not investigated.

Using this approach, optimization of the rectum parameters consists of a coarse grid search for the set [dose limit, penalty, volume] followed by linear interpolation and a fine grid search to yield the acceptable limit of RV50 as described in the preceding section. If required, the bladder, which does not affect target coverage as much as the rectum, is simply varied as a [dose limit, penalty, volume] set for a scaled percentage change until no more than 60% of the bladder volume is covered by 50% of the prescribed dose (BV50). If the bladder optimization affects the rectum RV50 by more than 2%, then the rectum optimization is repeated. The maximum dose limits to the rectum and bladder can be adjusted by changing their corresponding set [dose limit, penalty] by a preset increment of [–1,+10] for each percentage point above the limit. For example, if the bladder set [dose limit, penalty] of [100,5] resulted in a maximum bladder dose 2% above the acceptable limit, the next iteration would use the set of [98,25]. Target optimization consists of a grid search for the dose minimum penalty and dose maximum penalty [penalty $d_{\text{min}}$, penalty $D_{\max}$] sets shown in Table III.

**Table III:** Target penalty grid search. All combination values are tested and the set [Dmin,$D_{\max}$], which produces the highest target coverage, is used for critical structure optimization. Chosen target dose minimum penalty ($D_{\min}$ P): 50 and 20. Chosen target dose maximum penalty ($D_{\max}$ P): 50,100,150.

	$D_{\max}P=50$	$D_{\max}P=100$	$D_{\max}P=150$
$D_{\min}P=50$	[50,50]	[50,100]	[50,150]
$D_{\min}p=20$	[20,50]	[20,100]	[20,150]

The “optimum” set of target input parameters consists of the one that generates the best target coverage while maintaining the limits on the critical structures. We have found that the target volume receiving 100% of the dose (V100) is a sensitive index to select the optimum input parameters. Only the input parameter values that generate the highest improvement in V100 are used to re‐optimize the critical structures.

## RESULTS AND DISCUSSION

**Figure 3:**
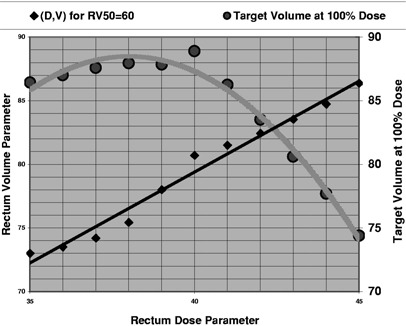
For a fixed rectum volume penalty, the corresponding dose and volume parameters (*D,V*) exhibit a linear relationship in order to produce a rectum DVH with a rectum dose of 50% to 60% of the rectum volume. Each parameter set produces a change in the target volume that receives 100% of the target dose.

The last phase of our optimization is to perform a limited search for those parameters that yield the best target coverage while maintaining the limits on the critical structures. Since the rectum set [dose limit, penalty, volume] has been the most important factor in determining the final target coverage, the final individual parameter search is limited to the rectum volumetric constraints. If the rectum penalty is held constant, the rectum dose as demonstrated by the data shown for a sample case in Fig. 3 to be a linear function of the rectum volume for a plan that is at the limit of acceptability as defined by a point on the rectum DVH. This greatly reduces the number of iterations. Using only a few points to generate a linear fit, the rest of the points are quickly evaluated to determine the best target coverage.

The rectum penalty is next changed by ±5% and the process is repeated. If the first few test points do not improve target coverage, the process stops. However, if the target coverage improves, the rectum penalty is varied again and the rectum dose limit and rectum volume evaluated. The algorithm ends when changes in rectum penalty do not improve target coverage. In extreme cases where acceptable plans cannot be generated the user may manually alter the fixed parameters and repeat the automated planning.

The algorithm computed deterministic input parameters are independent of the planner. These parameters generated better than acceptable plans. Dose distributions and DVHs were used to assess and compare the automated plans with corresponding manual plans. Analysis of DVHs showed that the V95 of PTV coverage improved by an average of 4.3% from that in plans generated without using the algorithm while still meeting the clinical acceptability criteria for critical structures, as shown in Table IV. The algorithm should also prove useful in dose escalation studies where it becomes increasingly difficult to maintain dose and volume limits to critical structures and the required subtle changes in the input parameters are harder to determine manually.

**Table IV:** Improvement in V95 treated to 95% dose using automated optimization compared to cases with input parameters manually selected by various planners.

Case	$V95_{\text{man}}$	$V95_{\text{opt}}$	Percent change
1	91.0	98.5	8.3
2	97.7	99.0	1.3
3	95.5	99.2	3.8
4	98.3	98.8	0.5
5	90.2	97.1	7.7
Mean			4.3

The concept of individual patient criteria for acceptability should be considered whenever reviewing patient results. If generic criteria are used for all patients, then a patient with a small overlap volume treated with a minimally acceptable plan could be underdosed in the clinical target volume. Furthermore, any single value nominal treatment “dose” for 3D plans may be inappropriate without a DVH. Two acceptable plans normalized to the same maximum dose may have similar V95 PTV values but vary significantly in the PTV covered by 100% of the prescribed dose.

Additional studies will be required to validate our findings and determine the usefulness of the algorithm on a large scale. At this time we have limited the search to a small interval for a limited number of cases. Testing will be required to determine if the algorithm converges on a local or absolute maximum and if it is of any clinical significance.^13^ Further evaluation is also needed to confirm that optimized plans at the limit of critical structure acceptability do not significantly increase complication rates. Since many plans can be generated that have a common point for a critical structure DVH, a detailed comparison of entire curves may be necessary to compare plans rather than individual points.

The parameter sequence, search categories, and computations can be facilitated by any readily available computational aid. We have used an EXCEL table augmented with a Visual Basic Application to perform some of the tasks. Obviously the optimum situation would be to implement the algorithm as part of the IMRT treatment planning system so that “optimized” plans could be generated automatically.^14^ Artificial intelligence techniques such as a rule‐based expert system could replace the human interface to the treatment planning system.

The methods described can be applied to any treatment planning system that requires the user input for selection of optimization parameters, especially if that system incorporates a similar inverse planning algorithm. Following our procedure, initial values of all the input parameters are determined based on manual planning experience for an anatomical site with standard fields. The various parameters are grouped into several categories, including those that will not change (fixed), those that can vary together (sets), those that can only have predetermined values (discrete), and those that can vary continuously over a range (individual). The number of free variables should be reduced as much as possible. After evaluating a few values for a parameter in the neighborhood of its initial value with a grid search interpolation techniques can be used to determine the exact value. It is very helpful to formulate relationships between parameters to limit the number of values to be tested. A simple but clinically relevant function, such as target coverage or dose uniformity, can be used to compare plans. Finally, there should be a predetermined goal to end the process.

Optimization is a relative process with numerous compromises. Gains in one area, such as improved target coverage, are often accompanied by undesirable effects such as increased dose to a critical structure. A mathematical recipe can facilitate plan evaluation but cannot replace experienced clinical judgment.

## CONCLUSIONS

An algorithm has been developed to assist in IMRT prostate treatment planning. Individual goals for target coverage are calculated for each case. The algorithm provides a comprehensive method to analytically select input parameters that result in improved acceptable plans based on target coverage and limits to critical structures. Optimization is also performed on the rectum volume parameters to maximize target coverage. Several cases planned automatically with input parameters that were calculated using the algorithm showed a significant improvement in target coverage when compared to manual plans. Typical manual acceptable plans can often be generated with fewer than ten trial input parameter values, but automated plans may require 20 to 30 iterations. However, quick random trials to determine if manual plans can be improved are inconclusive, and a thorough exhaustive analysis can easily exceed the number of automated planning iterations.
